# A Case of a CRTC1::TRIM11 Cutaneous Tumor With Venous and Lymphatic Invasion and Lymph Node Metastasis

**DOI:** 10.7759/cureus.83821

**Published:** 2025-05-09

**Authors:** Yoshimi Miyagi, Hideto Senzaki, Ikuma Kato, Yuko Kakuda, Keisuke Goto

**Affiliations:** 1 Department of Pathology, Osaka Saiseikai Nakatsu Hospital, Osaka, JPN; 2 Division of Pathology, Shizuoka Cancer Center, Shizuoka, JPN; 3 Department of Diagnostic Pathology and Cytology, Osaka International Cancer Institute, Osaka, JPN

**Keywords:** crtc1, crtc1::trim11 cutaneous tumor, cutaneous melanocytic tumor, melanocytoma, trim11

## Abstract

A *CRTC1::TRIM11* cutaneous tumor (CTCT) represents a novel and rare entity in dermatological oncology. We report a case of a 29-year-old Vietnamese woman who presented with a nodule on her right thigh and underwent surgical resection. Initially, a presumptive diagnosis of a fibrohistiocytic tumor was made based on histological features and immunohistochemical results, which were negative for S-100 and positive for CD68. However, further consultation revealed additional immunohistochemical findings: diffuse positivity for S-100, SOX10, and TRIM11, as well as focal positivity for HMB45 and MelanA. Fluorescence in situ hybridization showed no *EWSR1* gene rearrangement. Consequently, a final diagnosis of CTCT was established. Postoperative PET/CT scans suggested metastasis in the right inguinal lymph node, which was confirmed by excisional biopsy.

This case demonstrated partially invasive proliferation, venous and lymphatic invasion in the primary lesion, and lymph node metastasis, providing histological evidence of malignancy. Surgical resection is considered the primary treatment approach, but standardized protocols for adjuvant therapy have yet to be established. Targeted therapies against the *CRTC1::TRIM11* fusion gene may offer novel and more effective treatment options, and further research is warranted.

## Introduction

A *CRTC1::TRIM11* cutaneous tumor (CTCT) represents a novel and rare entity in the field of dermatological oncology. This newly recognized disease was included for the first time in the 2023 beta version of the fifth edition of the WHO Classification of Skin Tumours [1]. To our knowledge, there are 11 references in the English literature, reporting a total of 49 cases [2-12]. Most cases follow an indolent course; however, five aggressive cases involving recurrence, regional lymph node metastasis, or distant metastasis have been documented to date. We herein report a new case of CTCT characterized by venous and lymphatic invasion in the primary lesion and regional lymph node metastasis. This case is the 50th CTCT diagnosis and the 6th aggressive case worldwide, to the best of our knowledge. We present this case alongside a literature review and discuss the characteristics, diagnostic pitfalls, prognosis, and treatment strategies for this tumor.

## Case presentation

Clinical findings

**Figure 1:**
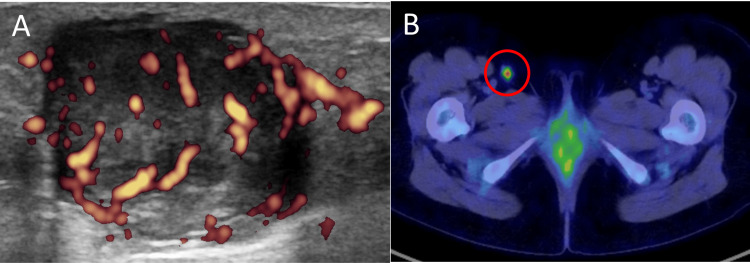
Imaging examination (A) The ultrasound examination revealed a well-circumscribed 3-cm subcutaneous mass with internal blood-flow signal. (B) Postoperative PET/CT suggested a metastasis in the right inguinal lymph node (SUV=4.3), circled in red. PET/CT: positron emission tomography/computed tomography; SUV: standardized uptake value

A 29-year-old Vietnamese woman residing in Japan without any remarkable past history or family history noticed a nodule on the posterior aspect of her right thigh two years ago, which was slowly growing. An ultrasound examination at our hospital revealed a well-circumscribed 3-cm subcutaneous mass with internal blood flow signals (Figure 1A). At the time of presentation, the patient had just discovered her pregnancy. Tumor resection was delayed and performed approximately one year after the initial visit, following childbirth and weaning. Postoperative positron emission tomography/computed tomography (PET/CT) showed findings suspicious for metastasis in the right inguinal lymph node (Figure 1B), which was confirmed by excisional biopsy (1/1 node positive); no distant metastases were suspected. The tumor had been completely resected during the initial surgery; nonetheless, an additional resection with a 2.0-cm margin was performed, along with further lymph node dissection, both of which were negative for tumor. At seven months of follow-up since the initial diagnosis, no local recurrence or nodal or distant metastasis has been observed.

Histopathology

**Figure 2:**
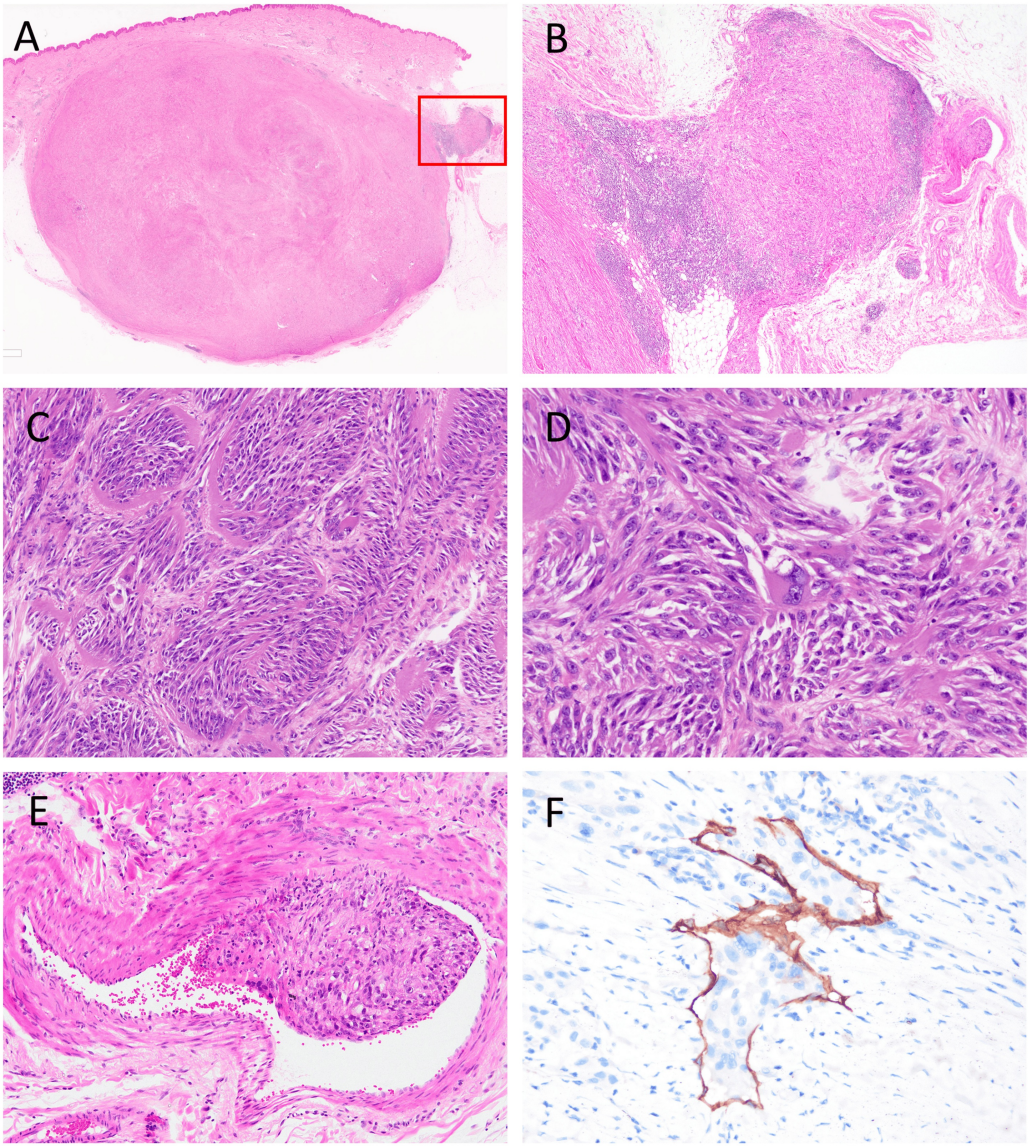
Histological findings of the primary lesion (A) A well-circumscribed, 2.5 cm nodular mass was observed from the subcutis to the dermis. (B) The tumor was not encapsulated, with partially invasive proliferation accompanied by lymphocytic infiltration; the region indicated by a red rectangle in (A). (C) The tumor consisted of spindle to epithelioid cells arranged in a fascicular and nested pattern, divided by fibrous septa. (D) Tumor cells had abundant pale cytoplasm and round or ovoid nuclei with prominent nucleoli. Multinucleated cells and giant cells were focally observed. (E) Venous invasion was observed. (F) Multiple lymphatic invasions were confirmed by D2-40 immunohistochemistry.

A well-circumscribed, 2.5-cm nodular mass was observed, extending from the subcutis to the dermis (Figure 2A). The mass was surrounded by a fibrous rim but lacked encapsulation, showing partially invasive proliferation accompanied by lymphocytic infiltration (Figure 2B). The tumor was composed of spindle to epithelioid cells arranged in fascicular and nested patterns, separated by fibrous septa (Figure 2C). The tumor cells exhibited abundant pale cytoplasm, round to ovoid nuclei, and prominent nucleoli, with few mitotic figures noted. Multinucleated and giant cells were focally present (Figure 2D). While neither necrosis nor perineural invasion was identified, histological examination revealed the presence of venous and lymphatic invasion (Figures 2E, 2F).

**Figure 3:**
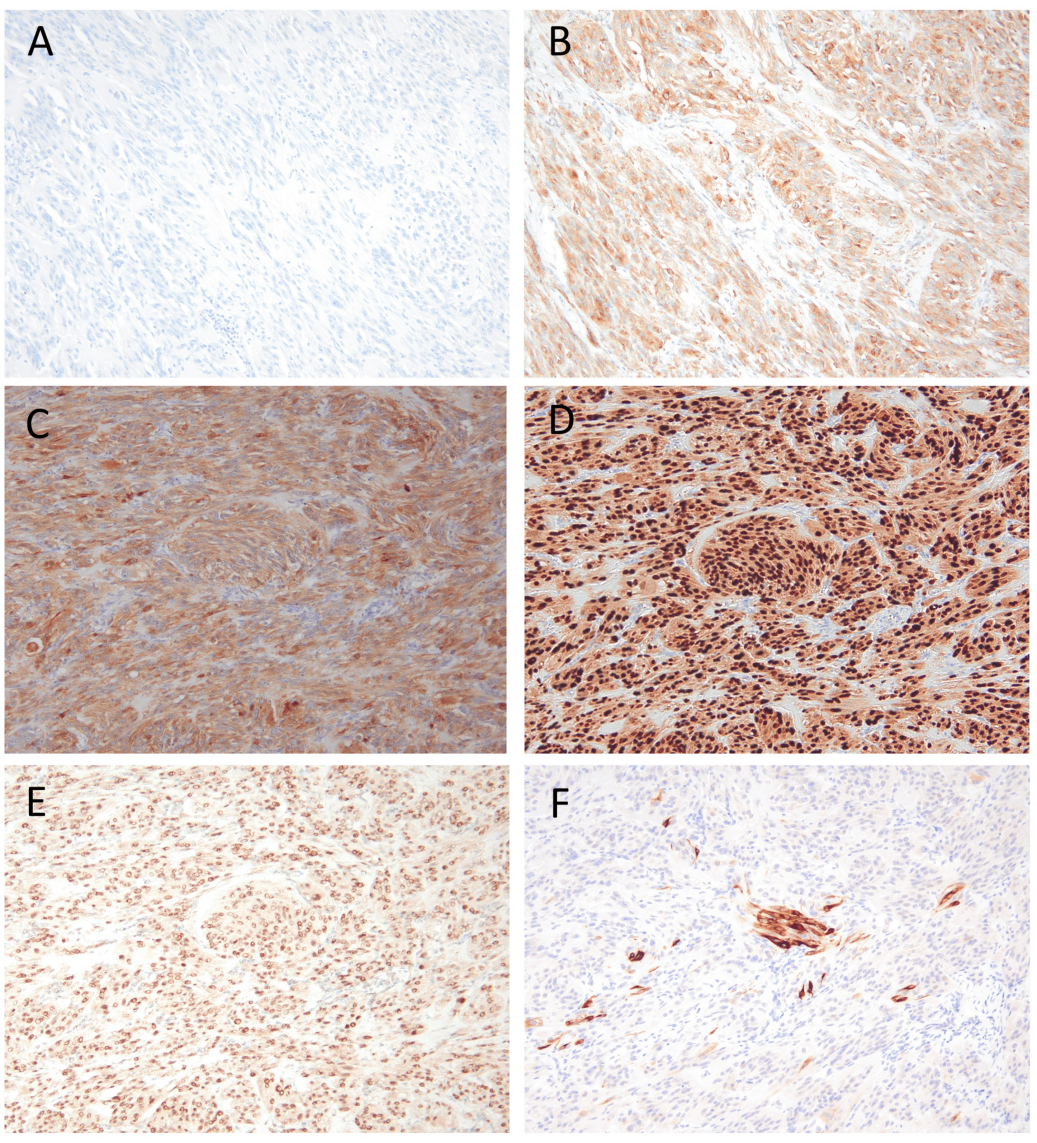
Immunohistochemical findings At the primary institution, S-100 was negative (A), and CD68 was positive (B). At the consulting facility, the tumor showed diffusely positive for S-100 (C), SOX10 (D), and TRIM11 (E), while focally positive for Melan A (F), which are typical findings of CTCT. CTCT: *CRTC1::TRIM11* cutaneous tumor

Initial immunohistochemical analysis revealed negativity for S-100, AE1/AE3, α-SMA, desmin, and CD34 but positivity for CD68 (Figures 3A, 3B), suggesting a presumptive diagnosis of a fibrohistiocytic tumor. However, further diagnostic consultation through the Japanese Society of Pathology and the National Cancer Center revealed different findings. The tumor exhibited diffuse positivity for S-100, SOX10, MITF, TRIM11, and CD68, along with focal positivity for HMB45, MelanA, and CD99, while being negative for ALK (Figures 3C-3F). Fluorescence *in situ* hybridization (FISH) for *EWSR1* gene rearrangement was also negative. Based on these findings, a final diagnosis of CTCT was established.

**Figure 4:**
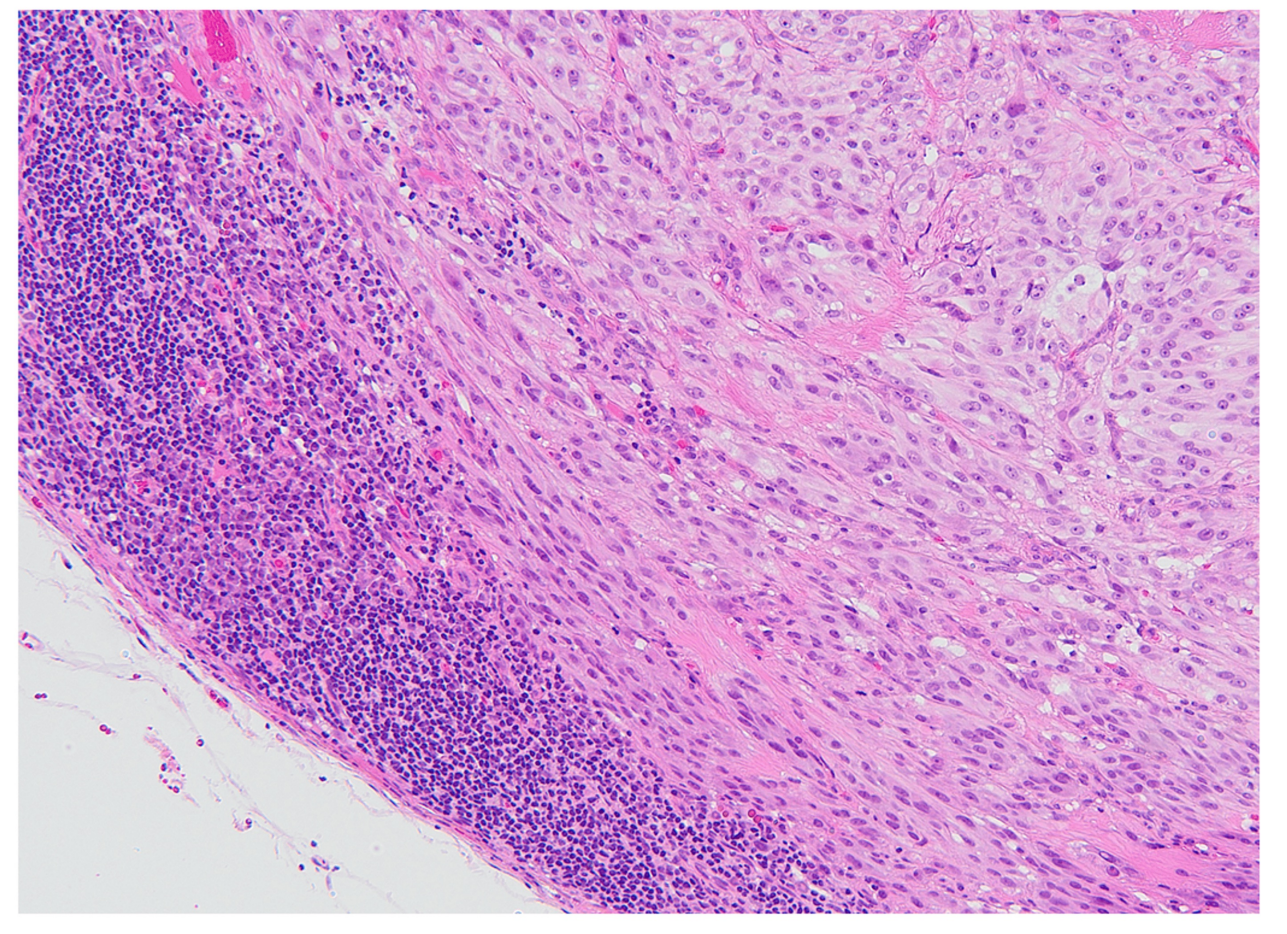
Histological findings of the metastatic lesion The histological features of the metastatic lesion in the right inguinal lymph node were consistent with those of the primary lesion.

An excisional biopsy of the right inguinal lymph node confirmed a single 7-mm metastatic lesion with histological features consistent with the primary tumor (Figure 4).

## Discussion

CTCT represents a novel and rare entity in the field of dermatological oncology. This disease was first included in the beta version of the fifth edition of the WHO Classification of Skin Tumours in 2023 [1]. Tracing its history, Cellier et al. first reported five cases of primary intradermal nodular unpigmented tumors with a melanocytic immunophenotype associated with a *CRTC1-TRIM11* fusion in 2018 [2]. They termed these tumors “cutaneous melanocytoma with *CRTC1-TRIM11* fusion.” In 2019, Bontoux et al. reported a case that was deeply located, infiltrative, and exhibited recurrence and metastasis [3]. They interpreted the disease as a clear cell sarcoma (CCS) with a novel *CRTC1-TRIM11* fusion rather than a melanocytoma. Subsequently, Kashima et al. reported a case and argued that the disease should be distinguished from CCS [4]. In 2022, Hanna et al. reported 41 cases, including 9 previously documented ones [8]. Among the 32 new cases they described, one had a recurrence, and another exhibited multiple lymph node metastases. Their report significantly contributed to the recognition of this disease as a distinct entity. Additional cases have been gradually reported. To date, including our case, a total of 50 cases have been documented, 6 of which are classified as aggressive.

Due to the typically well-circumscribed margins and slow-growing nature of CTCT, it may be clinically misdiagnosed as a benign lesion, potentially delaying the initiation of appropriate treatment. Histologically, CTCT often presents as a well-demarcated, unencapsulated nodule with a pushing border [1]. Although encapsulation has been reported in two cases, the capsule appears thin and incomplete rather than thick and circumferential [6]. Conversely, features such as loosely infiltrative nests and epidermal involvement have also been described [2,7]. In the present case, a partially invasive growth pattern accompanied by lymphocytic infiltration was observed (Figure 2b). Given that focal infiltration can occur, careful assessment of tumor margins, using multiple levels or deeper sections-may be warranted.

Immunohistochemically, the diffuse positivity rate in CTCT was 100% for SOX10, 44 % for S100, 9 % for Melan-A/Mart-1, 4 % for HMB-45, and 94 % for TRIM11, respectively [8]. In the present case, S-100 (Nichirei rabbit polyclonal) was negative at the primary institution, while S-100 (Dako rabbit polyclonal) was positive at the consulting facility. At the primary institution, positive staining was confirmed in both the external control on the same slide and internal controls, such as melanocytes and Langerhans cells in the epidermis, supporting the conclusion that the tumor's S-100 negativity was a true negative result rather than a technical error. This discrepancy highlights the potential variability in immunoreactivity depending on the antibody clone used, which can lead to diagnostic pitfalls. In cases where CTCT is suspected and S-100 staining appears negative, it is critical to verify the antibody clone employed. Using an alternative clone, such as the Dako rabbit polyclonal antibody, may yield a positive result and help avoid diagnostic errors. Also, CD68 (KP1) was positive at both institutions. In the literature, only four cases commented on CD68 expression, and all previous cases were negative [5]. The significance of CD68 positivity remains unclear; however, we believe that CD68 positivity should not exclude the diagnosis of CTCT. In addition to important differential diagnoses, such as CCS and malignant melanoma, fibrohistiocytic tumors should also be considered as potential differentials. As for the molecular features, demonstration of *CRTC1::TRIM11* fusion is necessary for the diagnosis [1]. Methods to demonstrate the *CRTC1::TRIM11* fusion include RNA sequencing, fluorescence in situ hybridisation (FISH), chromogenic in situ hybridization (CISH), reverse transcription-polymerase chain reaction (RT-PCR), and next-generation sequencing. However, these tests may not be feasible in all hospitals due to limitations in equipment, personnel, and cost. Consequently, immunodetection of overexpressed TRIM11, which reflects overexpression of the fusion product, has demonstrated a high positivity rate of 94%. We believe it may serve as a potentially applicable surrogate for these molecular diagnostic tests.

CTCT is generally perceived as a tumor with a relatively indolent nature; however, this case demonstrated venous and lymphatic invasion in the primary lesion, as well as lymph node metastasis, providing histological evidence of malignancy. The proportion of aggressive cases, including our case, is 12% (6/50) (Table 1). 

**Table 1:** Clinical features of aggressive cases NR: not reported, NA: not applicable, PD: progressive disease

Case	Age/Sex	Location	Recurrence or metastasis site (months after initial diagnosis)	Histological evidence	Pharmacotherapy	Outcome	Reference
1	31/F	Right arm	Recurrence, lung, axillary lymph node (all 156 months)	Presence (only recurrence)	Not administered	NA	[3]
2	NR	NR	Recurrence (6 months)	Presence	Not administered	NA	[8]
3	NR	Hand	lymph node (near elbow 1/1, axillary 3/36) (13 months)	Presence	Not administered	NA	[8]
4	30/M	Right hip	Inguinal lymph node (18,26,30 months), lung (36 months)	Presence (only lymph nodes)	Administered (six cycles of interferon, toripalimab, and etoricoxib)	PD	[9]
5	5/F	Right upper arm	Recurrence, lung, lymph node (upper arm and axilla) (all 19 months)	Presence (only lymph nodes)	Administered (six months of pembrolizumab)	PD	[7,11]
6	29/F	Right thigh	Inguinal lymph node (1/2) (0 months)	Presence	Not administered	NA	This case

The period after the initial diagnosis to recurrence or metastasis ranges widely from 0 to 156 months, highlighting the need for long-term follow-up. As for treatment, the presence of aggressive cases suggests the necessity of local excision. There have been two reports to date that specify the surgical margin distance for primary lesions. One of these reports includes five cases, all of which were excised with margins of 1.5 to 2.0 cm [2]. In all cases, no recurrence was observed. The other report describes a case where an initial margin of 0.5 cm was excised, followed by an additional 1.0 cm margin [7,11]. However, recurrence occurred in this case. There are reports in the literature indicating that for malignant soft tissue tumors in general, the recurrence rate for high-grade malignant soft tissue tumors is 7-13% when excised with a 2 cm margin of healthy tissue. In contrast, for low-grade malignant soft tissue tumors, the recurrence rate is 10% when excised with a 1 cm margin of healthy tissue [13]. Another study reported histological invasion extending up to 2.3 cm beyond the invasive findings seen on imaging [14]. It recommends securing a surgical margin of 2 cm beyond the tip of invasive findings on imaging, which could achieve a 98% rate of negative margins. 

In previous reports, three cases were found to have distant metastases, all of which involved the lungs [3,9,11]. However, there have been no cases where distant metastatic lesions were confirmed histologically, nor any reports of lymphvascular invasion identified in the primary lesion. This case is the first in the world to demonstrate venous and lymphatic invasion in the primary lesion, providing histological evidence that this tumor has the potential for distant metastasis. Among the cases with distant metastases to the lungs, two have undergone subsequent pharmacotherapy. One received six cycles of interferon, toripalimab, and etoricoxib [9], while the other underwent six months of pembrolizumab [11]. In both cases, the treatment outcome was progressive disease (PD). The absence of clinical response to interferon, immune checkpoint inhibitors (toripalimab, pembrolizumab), and COX-2 inhibition (etoricoxib) may reflect the unique molecular pathology of the *CRTC1::TRIM11* cutaneous tumor. This fusion-driven neoplasm may exhibit an immune-cold phenotype with low tumor mutational burden, making it less amenable to immune-based therapies. This therapeutic resistance may be explained by the molecular consequences of the *CRTC1::TRIM11* fusion gene, which results from a translocation involving the *CRTC1* transcriptional coactivator and the *TRIM11* ubiquitin E3 ligase. This fusion leads to the dysregulation of key cellular processes such as proliferation, apoptosis, and differentiation [15]. Inhibition of the associated signaling pathways or suppression of the fusion gene expression may contribute to the development of more effective therapeutic strategies. Elucidation of the underlying molecular mechanisms remains an important challenge for future research. Although radiation therapy has not yet been reported in CTCT, it may offer clinical benefit in cases of local recurrence. Whether radiation can meaningfully impact the fusion-driven oncogenic signaling remains unclear and warrants further investigation.

## Conclusions

This case confirms the malignant potential of CTCT, as evidenced by histologically proven venous and lymphatic invasion in the primary lesion and lymph node metastasis, thereby underscoring the need for long-term surveillance. Although wide excision with a 2-cm margin is regarded as the standard approach, pharmacological treatment options have yet to be established. Further investigation into the biological functions of *CRTC1::TRIM11* fusion and its therapeutic implications is warranted to address the unmet clinical needs in CTCT.
